# Covalency of hydrogen bonds in liquid water can be probed by proton nuclear magnetic resonance experiments

**DOI:** 10.1038/ncomms9318

**Published:** 2015-09-15

**Authors:** Hossam Elgabarty, Rustam Z. Khaliullin, Thomas D. Kühne

**Affiliations:** 1 Department of Chemistry, Dynamics of Condensed Matter, University of Paderborn, Warburger Street 100, D-33098 Paderborn, Germany; 2 Department of Chemistry, University of Zurich, Winterthurerstrasse 190, CH-8057 Zurich, Switzerland; 3 Department of Chemistry, McGill University, 801 Sherbrooke Str. West, Montreal, Quebec, Canada H3A 0B8; 4 Paderborn Center for Parallel Computing and Institute for Lightweight Design with Hybrid Systems, Warburger Street 100, D-33098 Paderborn, Germany

## Abstract

The concept of covalency is widely used to describe the nature of intermolecular bonds, to explain their spectroscopic features and to rationalize their chemical behaviour. Unfortunately, the degree of covalency of an intermolecular bond cannot be directly measured in an experiment. Here we established a simple quantitative relationship between the calculated covalency of hydrogen bonds in liquid water and the anisotropy of the proton magnetic shielding tensor that can be measured experimentally. This relationship enabled us to quantify the degree of covalency of hydrogen bonds in liquid water using the experimentally measured anisotropy. We estimated that the amount of electron density transferred between molecules is on the order of 10  m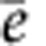 while the stabilization energy due to this charge transfer is ∼15 kJ mol^−1^. The physical insight into the fundamental nature of hydrogen bonding provided in this work will facilitate new studies of intermolecular bonding in a variety of molecular systems.

## Abstract (web summary)

Covalency is a fundamental concept in chemical bonding, but experimentally it is not possible to measure the degree of covalency of a particular bond. Here, the authors report a model to link the covalency of hydrogen bonds in water with the anisotropy of the magnetic shielding tensor in the proton NMR.

## Graphical abstract

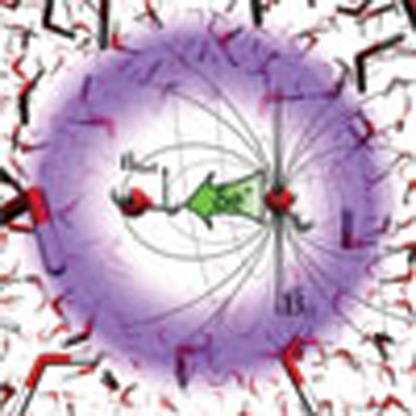

From its early days, NMR spectroscopy has been instrumental in the study of liquid water^1^,^2^. The magnetic shielding tensor, which relates secondary induced electronic magnetic fields to an external static magnetic field, is at the heart of NMR spectroscopy. The shielding tensor is characterized by the three components in the principal axes frame *σ*_*X*_≤*σ*_*Y*_≤*σ*_*Z*_ or, alternatively, by the isotropic part *σ*_iso_=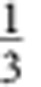
(*σ*_*X*_+*σ*_*Y*_+*σ*_*Z*_), the anisotropy Δ=*σ*_*Z*_−*σ*_iso_, and the asymmetry *η*=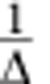
(*σ*_*Y*_−*σ*_*X*_)^3^. However, because of the random orientation and motion of molecules in the liquid phase, only the isotropic part of the tensor can be directly measured by experiment. Nevertheless, even the isotropic ^1^H shielding can provide valuable information: it has been long established that *σ*_iso_ decreases on the formation of hydrogen bonds (HBs)^4^,^5^,^6^.

A quantitative investigation between the other components of the shielding tensor and the HB geometry in liquid water^7^,^8^ and small cluster models^4^,^9^,^10^ has been made possible by advances in electronic structure theory^11^,^12^,^13^,^14^,^15^,^16^. Moreover, a recently reported elegant experimental approach has allowed the indirect measurement of the ^1^H shielding anisotropy in liquid water via its contribution to spin relaxation rates^17^. Combined with first principles calculations, the new experimental data^8^ have underscored previous findings that some of the tensor components are more sensitive to HB interactions than the average *σ*_iso_ (refs 4, 18).

In this work, we reveal a quantitative relationship between the calculated components of the ^1^H magnetic shielding tensor and the degree of covalency of HBs in liquid water^19^,^20^. The covalent component of intermolecular bonds is commonly defined as the strength of donor–acceptor orbital interactions between molecules^21^. Covalency, like many other individual fundamental mode of binding (for example, frozen electrostatics, polarization), cannot be measured, even in principle, by experiment; only the total strength of binding is measurable. Nevertheless, similar to various other unmeasurable auxiliary concepts (for example, wave function in quantum mechanics), covalency is a fundamental, theoretically well defined and physically meaningful quantity that is widely used by chemists to investigate the nature of intermolecular bonding. Furthermore, it is widely accepted that donor–acceptor interactions between molecules in liquid water affect its directly measurable structural, spectroscopic and chemical properties.

## Results

### Decomposition analysis and NMR shielding tensor

To quantify the strength of covalent interactions in a HB, we employed the decomposition analysis for condensed phase systems based on absolutely localized molecular orbitals (ALMO DA)^21^,^22^ within Kohn–Sham density functional theory^23^. The ALMO DA makes this feat possible by first filtering out frozen electrostatic and polarization effects from the total many-body intermolecular binding energy, and then splitting the remaining covalent component into two-body terms, each corresponding to an individual HB. The ALMO approach allows to define two scales to measure the strength of covalent interactions: the amount of electron density transferred from the occupied orbitals on molecule A to the virtual orbitals on molecule B (Δ*Q*_A→B_), and the energetic stabilization due to this charge transfer (Δ*E*_A→B_). These two-body terms are obtained self-consistently and include cooperativity effects, which are essential for a correct description of the HB network in liquid water^24^,^25^,^26^,^27^.

All ensemble averaged components of the NMR shielding tensor (Table 1), geometric and ALMO DA descriptors of HBs were calculated from density functional theory-based *ab initio* molecular dynamics (MD) simulations performed to represent liquid water at ambient conditions (see Methods for details). The computed values are close to the experimentally measured absolute shielding values at 27 °C (*σ*_iso_=25.7 p.p.m. and Δ=18.3 p.p.m., respectively)^17^. We followed the common practice of denoting the average of *σ*_*X*_ and *σ*_*Y*_ components of the axially nearly symmetric NMR tensors (*η*≈0) as the perpendicular shielding component *σ*_⊥_. It is easy to see that *σ*_⊥_ is directly related to the anisotropy: *σ*_⊥_=*σ*_iso_−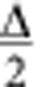.

### Correlation between *σ* components, HB covalency and geometry

**Figure 1:**
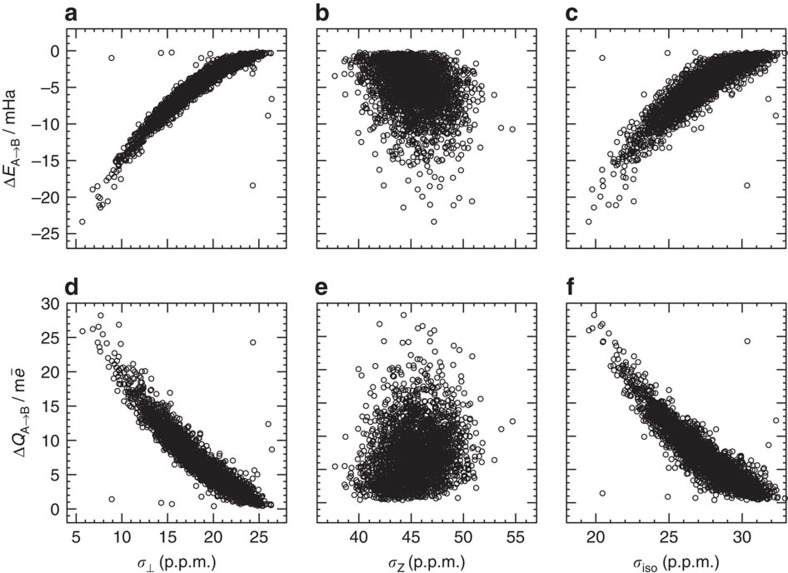
Relationship between the electronic descriptors and NMR characteristics of HBs. Δ*E*_A→B_ and Δ*Q*_A→B_ as a function of *σ*_⊥_, *σ*_*Z*_ and *σ*_iso_, respectively.

Figure 1a,d show that *σ*_⊥_ exhibits a striking correlation with the strength of electron density transfer as measured on both charge and energy scales. By comparison, the correlation between *σ*_iso_ and the strength of charge transfer (Fig. 1c,f) is weaker, because *σ*_iso_ is just the strongly correlated *σ*_⊥_ component plus the noise from *σ*_*Z*_ (Fig. 1b,e).

The presence of the scattered points in Fig. 1 that do not follow the general trend is a manifestation of the complex nature of the HB network, in which some hydrogen atoms do not form HBs and some interact strongly with more than one electron donor. Describing such configurations in detail is beyond the scope of the present work and will be presented elsewhere. Here we just would like to note that including or excluding the outliers does not change our statistical results, models or conclusions significantly (see Methods).

**Figure 2:**
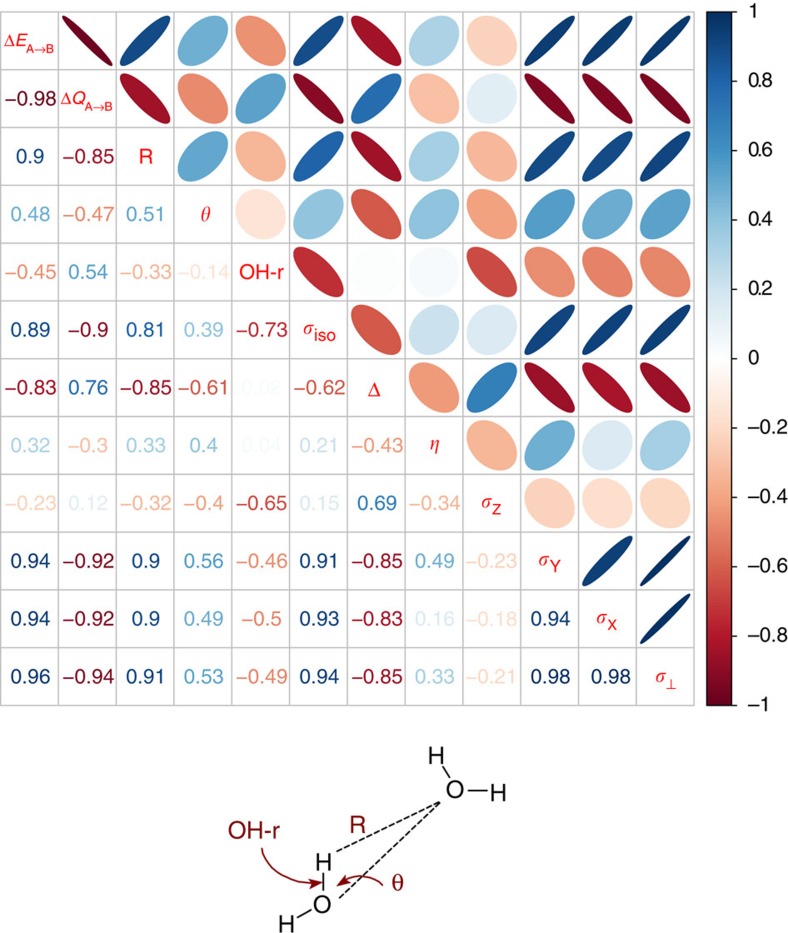
Correlation matrix between the ^1^H shielding tensor components and HB descriptors. The descriptors are shown on the main diagonal. The lower part of the matrix gives numerical values of the Pearson correlation coefficient *r* between a pair of variables. The upper part above the diagonal presents a symbolic representation of the same correlation: ellipses represent different degrees of correlation ranging from *r*=1 (straight line) to *r*=0 (perfect circle). The geometric descriptors are illustrated on the water dimer at the bottom, where OH-r is the covalent O-H bond length and *R* the HB length, while *θ* is the HB angle.

To obtain a more comprehensive view on HBs in liquid water we collected information on the correlation between electronic, NMR and geometric descriptors into a single matrix shown in Fig. 2. In addition to the key relationship established above, the correlation matrix clearly shows the well-known relationship between the HB length and *σ*_iso_. However, we found that the correlation of the HB length and *σ*_⊥_ is stronger. As in the case of the electronic descriptors, the correlation between the HB length and *σ*_iso_ is merely a manifestation of the underlying strong correlation with *σ*_⊥_ plus a noisy contribution from *σ*_*Z*_.

Δ*Q*_A→B_ and Δ*E*_A→B_ are also strongly correlated with the HB length, even though this correlation is somewhat weaker than that with *σ*_⊥_ because the HB length alone is insufficient to characterize a HB (that is, other geometric descriptors, such as the HB angle, should be taken into account). We note that no correlations were found for the electron transfer terms where the water molecule bearing the shielding tensor acts as an electron donor. This shows that the ^1^H shielding tensor is insensitive to electron transfer from the lone pairs of its own water molecule. There is no significant correlation of the HB angle with any individual shielding tensor component, except for a rather weak correlation with Δ. The HB angle alone does not appear to influence electronic terms either, in agreement with the insensitivity of Δ*Q*_A→B_ and Δ*E*_A→B_ to intermolecular rotations in water dimer^28^.

**Figure 3:**
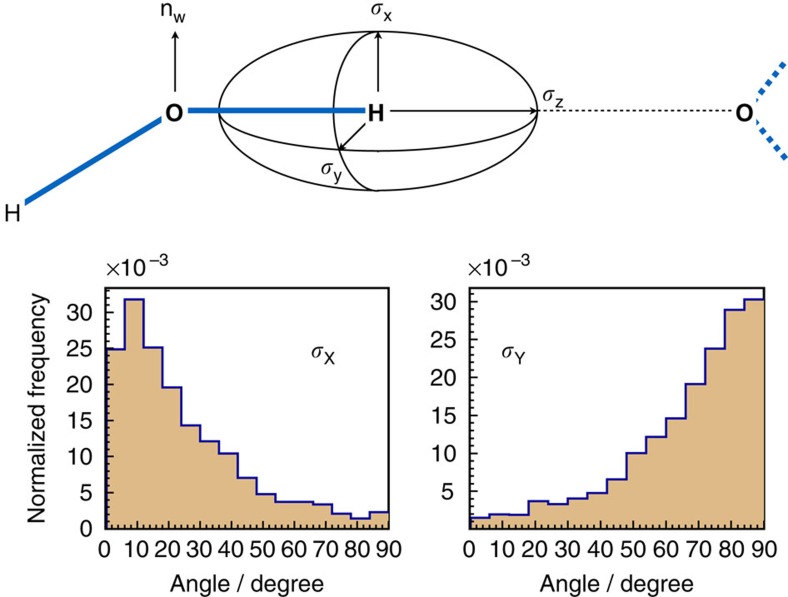
Spatial orienation of the ^1^H shielding tensor relative to the HB geometry. The ^1^H shielding tensor ellipsoid is rigidly fixed to the molecular frame of the HB donating water molecule. The longest axis of the ellipsoid (*σ*_*Z*_) always points along the HB. The angle between the normal to the water molecular plane (n_w_) and *σ*_*X*_ and *σ*_*Y*_ show that the former is parallel to n_w_, while the latter is orthogonal to it.

In addition to studying the shape of the shielding ellipsoid, we also examined its orientation relative to the water molecule. As in previous studies^29^, our simulations showed that the longest *Z* axis of the tensor points along the covalent O—H bond: the mean deviation angle between the longest axis and the covalent O—H bond is only 5±3° (Fig. 3, top). Moreover, despite the very low value of *η*, we find that the two short axes still exhibit a distinct spatial preference: the shortest *X* axis is normal to the plane of the water molecule, whereas the intermediate *Y* axis is coplanar with the molecule (Fig. 3, bottom). In other words, the shielding tensor is rigidly fixed to the water molecule. Previous reports have shown that the only non-zero off-diagonal elements of the shielding tensor are *σ*_*yz*_ and *σ*_*zy*_ in the Eckart frame^7^,^30^. This is consistent with our finding, as the transformation from the principal axes frame to the Eckart frame is merely a rotation around the *X* axis.

### Linear model that relates *σ*_⊥_ to the covalency of HBs

The physical basis underlying the correlation between the electronic and NMR terms (Fig. 1) can be understood by considering the dependence of both of these quantities on the HB length *R*. We found that *σ_x_* and *σ_y_* decrease linearly with *R*^−3^ over the entire HB length range in liquid water (see Supplementary Fig. 1). This strong negative correlation implies that the induced magnetic field from the electron donor molecule (a schematic depiction is given in Supplementary Fig. 2) is the major contributor to deshielding in the plane orthogonal to the HB—it accounts for 88% of the variance in *σ*_⊥_. The inverse cubic dependence also suggests that this field can be accurately represented as that of a magnetic dipole. That is, higher order terms in the multipole expansion (Supplementary Equation 1) are negligible. Thus, the *R*^−3^ dependence of *σ*_⊥_ enables one to write:


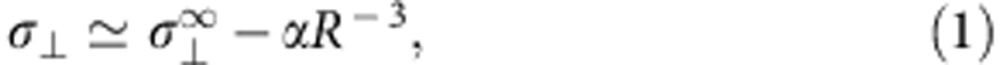


where *σ*_⊥_^∞^ characterizes an HB-free water molecule, and has a value of 33.7 p.p.m. as obtained by linearly extrapolating *σ*_⊥_ to *R*^−3^=0. The strength of donor–acceptor interactions between molecules decreases with the tails of the orbitals, that is, exponentially with distance^21^


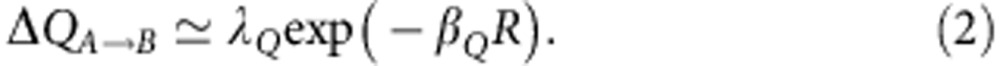


**Figure 4:**
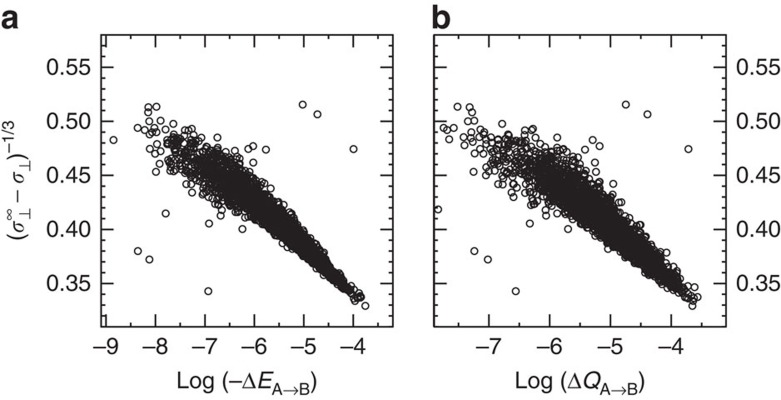
The derived linear model relating *σ*_⊥_ to the electronic descriptors as obtained by ALMO DA. Dependence between (*σ*_⊥_^∞^−*σ*_⊥_)^−1/3^ and: (**a**) log(−Δ*E*_A→B_), (**b**) log(Δ*Q*_A→B_).

The combination of the two equations predicts the following relationship between the charge-transfer term and perpendicular component of the NMR shielding tensor:


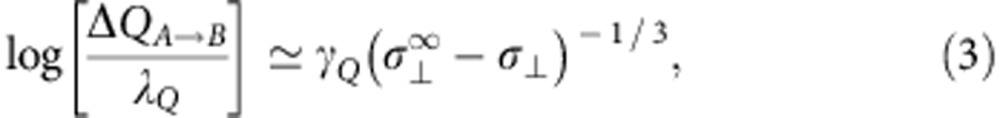


where *γ*_*Q*_=−*β*_*Q*_*α*^1/3^ is the dimensionless proportionality constant. A similar relationship can be written for the energy scale with *β*_*E*_, *γ*_*E*_, *λ*_*E*_ as parameters. Indeed, plotting both sides of equation (3) reveals clear linear trends (Fig. 4) with a Pearson coefficient of −0.94 for Δ*E*_A→B_ and −0.92 for Δ*Q*_A→B_. The values of the parameters that represent the best fit of the simulation data are *λ*_*Q*_=270.6 m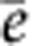, *λ*_*E*_=579.4 mHa, *γ*_*Q*_=− 19.86 and *γ*_*E*_=−24.07, respectively. It is worth noting that the *λ* coefficients should not be interpreted as the limiting values for *R*→0, but rather as statistical coefficients that best fit the linear trend over its limited range of validity.

While *σ*_⊥_ shows the strong *R*^−3^ dependence, the weak correlation between *σ*_*Z*_ and *R*^−3^ (Supplementary Fig. 1) indicates that the change in *σ*_*Z*_ results from the factors other than the dipolar field of the electron donor molecule. We found that a satisfactory regression model capable of predicting the behaviour of *σ*_*Z*_ should include at least three predictor variables: *R*^−3^, the HB angle *θ* and the covalent O—H bond length OH-r. The standardized regression coefficients of this model (Supplementary Table 1) show that *R*^−3^ and OH-r give opposite contributions to *σ*_*Z*_ largely cancelling each other out.

The established quantitative dependence between *σ*_⊥_ and the strength of intermolecular donor–acceptor bonding has one important implication: since *σ*_⊥_ can now be measured experimentally in liquids^17^, our model represents an indirect method for probing the covalent component of HBs. Table 2 presents our estimates of Δ*E*_A→B_ and Δ*Q*_A→B_ in liquid water based on a linear approximation to the regression model established here and the *σ*_⊥_ measured experimentally at different temperatures^17^ (Supplementary Note 1 for full analysis). As expected, the strength of the covalent component in HBs decreases with increasing temperature as more water molecules form distorted or even broken HBs with their neighbours.

We would like to emphasize that, unlike population analysis methods (for example, Mulliken, Löwdin), which partition the total electron density between molecules, the ALMO approach measures the reorganization of electron clouds on the formation of a HB^31^. This conceptual advantage of the ALMO method leads to the remarkable result that the fractional electron transfer Δ*Q*_A→B_ between water molecules in the liquid phase is only a few milli-electrons (Table 2). The reorganization of charge has a well-defined energy given by the Δ*E*_A→B_ term. Whereas it may seem unusual that so little charge transfer (7–10 m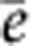) can stabilize a HB by 12–18 kJ mol^−1^ (equivalent to 19 eV per one transferred electron), it is consistent with simple theoretical estimates. Perturbation theory shows that the transfer energy per electron is equal to the energy gap between donating and accepting orbitals^28^. Since the energy gap between the most important donating and accepting orbitals on two molecules lies between 10 and 40 eV (virtual orbitals form almost a continuum of states), a value of 19 eV for the effective donor–acceptor gap is entirely reasonable.

To put the estimates of Δ*Q*_A→B_ into a context, we calculated that 4 m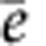 is transferred between molecules in the water dimer if the two molecules are at their equilibrium separation of 2.0 Å. However, Δ*Q*_A→B_ becomes 7.6 m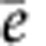 (that is, significantly closer to the value obtained for the ambient liquid) if the molecules in the dimer are separated only by 1.77 Å—the typical length of a HB in the liquid phase^27^.

It is important to note that the timescale of fluctuations in Δ*Q*_A→B_ and Δ*E*_A→B_ is the same as the timescale of intermolecular vibrations—hundreds of femtoseconds^22^,^32^,^33^,^34^, which is several orders of magnitude faster than the typical timescale of NMR spectroscopy. This implies that NMR measurements of covalency are capable of yielding only time-averaged values of Δ*Q*_A→B_ and Δ*E*_A→B_.

## Discussion

To summarize, we established a simple quantitative relationship between the perpendicular component *σ*_⊥_ of the axially nearly symmetric ^1^H magnetic shielding tensor and the degree of covalency of HBs in liquid water. Covalency was defined as the amount of electron density transfer between water molecules and the stabilization energy associated with it. The physical origin of this relationship is the field induced almost entirely by the magnetic dipole located on the neighbouring water molecule involved in the HB. The major implication of our findings is that this relationship provides the calibration necessary to quantify the covalency of HBs experimentally.

Recent advancements in measuring *σ*_⊥_ for liquid water^17^ enabled us to estimate that the average amount of charge transferred between the molecules on the formation of an average HB is on the order of 10 m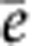, while the corresponding stabilization energy is estimated to be 15 kJ mol^−1^. From the practical perspective, using *σ*_⊥_ rather than *σ*_*X*_ or *σ*_*Y*_ offers an experimental advantage because *σ*_⊥_ can be determined as a linear combination of *σ*_iso_ and Δ and it is technically easier to measure the latter.

In contrast to *σ*_⊥_, the *σ*_*Z*_ component of the shielding tensor exhibits a complex dependence on the local environment around the proton. Its fluctuations cannot be explained only by the magnitude of the induced magnetic fields originating at the proton-accepting water molecule. Therefore, although it is trivial to measure *σ*_iso_ experimentally, the noisy contribution of *σ*_*Z*_ makes it unsuitable for predicting covalency.

## Methods

### Second generation Car–Parrinello *ab initio* MD simulations

*Ab initio* MD simulations of a periodic cubic cell with 128 light water molecules were performed at constant temperature (330 K to mimic nuclear quantum effects in liquid water at ambient conditions^35^) and density (0.9966, g cm^−3^) using the second generation Car–Parrinello method^36^,^37^. In this approach, the fictitious Newtonian dynamics of the original Car–Parrinello scheme^38^ is replaced with an improved coupled electron-ion dynamics that keeps electrons close to the instantaneous electronic ground state without defining a fictitious mass parameter. The superior efficiency originates from the fact that the iterative wave function optimization is fully bypassed. Thus, not even a single diagonalization step is required, while at the same time allowing for integration time steps up to the ionic resonance limit.

The energy and forces were computed using the mixed Gaussian-plane waves approach^39^, where the Kohn–Sham orbitals were represented by an accurate triple-*ζ* basis set with two sets of polarization functions (TZV2P)^40^, while plane-waves with cutoff of 400 Ry were used to represent the charge density. The BLYP exchange-correlation functional plus a damped interatomic potential to account for van der Waals interactions^41^ was employed. Previous works have shown that this set-up provides a realistic description of many important structural, dynamical and spectroscopic characteristics of liquid water, including the partial pair correlation functions, self-diffusion and viscosity coefficients, HB lifetime, vibrational spectra and magnetic shielding tensor components^14^,^15^,^27^,^42^,^43^. The system has been equilibrated at constant temperature and density for 30 ps before ten decorrelated snapshots separated by 1 ps were extracted.

### Computational analysis

The ALMO DA was performed for each snapshot using the same computational set-up as in ref. 22. The magnetic shielding tensors were computed using density functional perturbation theory^14^,^44^,^45^ that is based on the all-electron GAPW method^46^,^47^ and IGLO-III basis set^48^. All computations were performed using the QUICKSTEP and ALMO modules of the CP2K suite of programmes^49^. To keep our results consistent, we performed sampling over all protons including the outliers that are clearly visible in Figs 1 and 4. The only exception is the analysis presented in Fig. 2 (supported by Supplementary Fig. 1 and Supplementary Table 1). The data presented in Fig. 2 requires computing geometric characteristics of HBs. These characteristics can be computed only for well defined HBs. To define a HB, we used a geometric criterion that was derived from the two-dimensional (*R* versus *θ*) potential of mean force^43^,^50^.

## Additional information

**How to cite this article:** Elgabarty, H. *et al.* Covalency of hydrogen bonds in liquid water can be probed by proton nuclear magnetic resonance experiments. *Nat. Commun.* 6:8318 doi: 10.1038/ncomms9318 (2015).

## Supplementary Material

Supplementary Information Supplementary Figures 1-3, Supplementary Tables 1-2 and Supplementary Note 1

## Figures and Tables

**Table 1:** Calculated ensemble averaged shielding tensor components in p.p.m. for liquid water at ambient conditions.

	***σ***_iso_	**Δ**	***η***	***σ***_***X***_	***σ***_***Y***_	***σ***_***Z***_
mean	27.34	18.00	0.13	17.21	19.48	45.33
s.e.	0.04	0.06	0.001	0.07	0.07	0.05
s.d.	2.21	3.10	0.08	3.42	3.57	2.29

**Table 2:** Estimated average values for Δ*E*_A→B_ and Δ*Q*_A→B_ in liquid water at different temperatures.

**T./°C**	***σ***_**⊥**_**(p.p.m.)**	**Δ*****Q***_**A→B**_**/m** 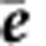	**Δ*****E***_**A→B**_**/mHa**
80	17.9	7.09 (7.02,7.17)	−4.68 (−4.63,−4.73)
27	16.6	8.71 (8.61,8.82)	−6.01 (−5.93,−6.08)
0	15.9	9.65 (9.53,9.78)	−6.80 (−6.71,−6.89)

The estimates are based on the experimentally determined *σ*_⊥_ (ref. 17) and the regression model given by Equation (3). Brackets represent the 95% C.I. of the regression model.

## References

[b1] Bloembergen N., Purcell E. & Pound R. Relaxation effects in nuclear magnetic resonance absorption. Phys. Rev. 73, 679–712 (1948).

[b2] Simpson J. & Carr H. Diffusion and nuclear spin relaxation in water. Phys. Rev. 111, 1201–1202 (1958).

[b3] Harris R. K. *et al.* Further conventions for NMR shielding and chemical shifts (IUPAC Recommendations 2008). Magn. Reson. Chem. 46, 582–598 (2008). 18407566 10.1002/mrc.2225

[b4] Ditchfield R. Theoretical studies of magnetic shielding in H2O and (H2O)2. J. Chem. Phys. 65, 3123–3133 (1976).

[b5] Konrat R., Tollinger M., Kontaxis G. & Kräutler B. NMR techniques to study hydrogen bonding in aqueous solution. Monatsh. Chem. 130, 961–982 (1999).

[b6] Grzesiek S. & Becker E. D. Hydrogen Bonding. eMagRes. (2011).

[b7] Pennanen T. S. *et al.* Nuclear magnetic shielding and quadrupole coupling tensors in liquid water: a combined molecular dynamics simulation and quantum chemical study. J. Am. Chem. Soc. 126, 11093–11102 (2004). 15339196 10.1021/ja048049i

[b8] Modig K., Pfrommer B. & Halle B. Temperature-Dependent hydrogen-bond geometry in liquid water. Phys. Rev. Lett. 90, 075502 (2003). 12633241 10.1103/PhysRevLett.90.075502

[b9] Chesnut D. & Rusiloski B. A study of NMR chemical shielding in water clusters derived from molecular dynamics simulations. J. Mol. Struct. 314, 19–30 (1994).

[b10] Limbach H.-H. *et al.* OHO hydrogen and bond geometries and NMR chemical and shifts: from equilibrium structures to geometric H/D isotope effects, with applications for water, protonated water, and compressed ice. Isr. J. Chem. 49, 199–216 (2009).

[b11] Gauss J. Calculation of NMR chemical shifts at second-order many-body perturbation theory using gauge-including atomic orbitals. Chem. Phys. Lett. 191, 614 (1992).

[b12] Gauss J. Effects of electron correlation in the calculation of nuclear magnetic resonance chemical shifts. J. Chem. Phys. 99, 3629 (1993).

[b13] Pickard C. & Mauri F. All-electron magnetic response with pseudopotentials: NMR chemical shifts. Phys. Rev. B 63, 1–13 (2001).

[b14] Sebastiani D. & Parrinello M. A new *ab Initio* approach for NMR chemical shifts in periodic systems. J. Phys. Chem. A 105, 1951–1958 (2001).

[b15] Sebastiani D. & Parrinello M. Ab-initio study of NMR chemical shifts of water under normal and supercritical conditions. Chem. Phys. Chem. 3, 675–679 (2002). 12503147 10.1002/1439-7641(20020816)3:8<675::AID-CPHC675>3.0.CO;2-O

[b16] Ochsenfeld C., Kussmann J. & Koziol F. Ab Initio NMR Spectra for molecular systems with a thousand and more atoms: A linear-scaling method. Angew. Chem. Int. Ed. 43, 4485–4489 (2004). 10.1002/anie.200460336 15317006

[b17] Modig K. & Halle B. Proton magnetic shielding tensor in liquid water. J. Am. Chem. Soc. 124, 12031–12041 (2002). 12358550 10.1021/ja026981s

[b18] Saitô H., Ando I. & Ramamoorthy A. Chemical shift tensor - the heart of NMR: Insights into biological aspects of proteins. Prog. Nucl. Magn. Reson. Spectrosc. 57, 181–228 (2010). 20633363 10.1016/j.pnmrs.2010.04.005 PMC2905606

[b19] Scheiner S. Hydrogen bonding. A theoretical perspective Oxford University Press (1997).

[b20] Grabowski S. J. What is the covalency of hydrogen bonding? Chem. Rev. 111, 2597–2625 (2011). 21322583 10.1021/cr800346f

[b21] Khaliullin R. Z., Cobar E. A., Lochan R. C., Bell A. T. & Head-Gordon M. Unravelling the origin of intermolecular interactions using absolutely localized molecular orbitals. J. Phys. Chem. A 111, 8753–8765 (2007). 17655284 10.1021/jp073685z

[b22] Khaliullin R. Z. & Kühne T. D. Microscopic properties of liquid water from combined ab initio molecular dynamics and energy decomposition studies. Phys. Chem. Chem. Phys. 15, 15746–15766 (2013). 23928575 10.1039/c3cp51039e

[b23] Kohn W. Nobel lecture: electronic structure of matter - wave functions and density functionals. Rev. Mod. Phys. 71, 1253–1266 (1999).

[b24] Frank H. S. Covalency in the Hydrogen bond and the properties of water and ice. Proc. Roy. Soc. A247, 481–492 (1958).

[b25] Luck W. A. P. The importance of cooperativity for the properties of liquid water. J. Mol. Struct. 448, 131 (1998).

[b26] Glendening E. D. Natural energy decomposition analysis: Extension to density functional methods and analysis of cooperative effects in water clusters. J. Phys. Chem. A 109, 11936–11940 (2005). 16366646 10.1021/jp058209s

[b27] Kühne T. D., Krack M. & Parrinello M. Static and Dynamical properties of liquid water from first principles by a novel Car–Parrinello-like approach. J. Chem. Theory Comput. 5, 235–241 (2009). 10.1021/ct800417q 26610101

[b28] Khaliullin R. Z., Bell A. T. & Head-Gordon M. Electron donation in the water-water hydrogen bond. Chem. Eur. J. 15, 851–855 (2009). 19086050 10.1002/chem.200802107

[b29] Pfrommer B. G., Mauri F. & Louie S. G. NMR chemical shifts of ice and liquid water: the effects of condensation. J. Am. Chem. Soc. 122, 123–129 (2000).

[b30] Vaara J., Lounila J., Ruud K. & Helgaker T. Rovibrational effects, temperature dependence, and isotope effects on the nuclear shielding tensors of water: a new 17O absolute shielding scale. J. Chem. Phys. 109, 8388–8397 (1998).

[b31] Khaliullin R. Z., Bell A. T. & Head-Gordon M. Analysis of charge transfer effects in molecular complexes based on absolutely localized molecular orbitals. J. Chem. Phys. 128, 184112 (2008). 18532804 10.1063/1.2912041

[b32] Fecko C. J. Ultrafast hydrogen-bond dynamics in the infrared spectroscopy of water. Science 301, 1698–1702 (2003). 14500975 10.1126/science.1087251

[b33] Kühne T. D. & Khaliullin R. Z. Electronic signature of the instantaneous asymmetry in the first coordination shell of liquid water. Nat. Commun. 4, 1450 (2013). 23385594 10.1038/ncomms2459

[b34] Kühne T. D. & Khaliullin R. Z. The nature of the asymmetry in the hydrogen-bond networks of hexagonal ice and liquid water. J. Am. Chem. Soc. 136, 3395–3399 (2014). 24521433 10.1021/ja411161a

[b35] Morrone J. & Car R. Nuclear quantum effects in water. Phys. Rev. Lett. 101, 017801 (2008). 18764152 10.1103/PhysRevLett.101.017801

[b36] Kühne T. D., Krack M., Mohamed F. R. & Parrinello M. Efficient and accurate Car–Parrinello-like approach to Born- Oppenheimer molecular dynamics. Phys. Rev. Lett. 98, 0664501 (2007). 10.1103/PhysRevLett.98.066401 17358962

[b37] Kühne T. D. Second generation Car–Parrinello molecular dynamics. Wiley Interdiscip. Rev. Comput. Mol. Sci. 4, 391 (2014).

[b38] Car R. & Parrinello M. Unified approach for molecular dynamics and density-functional theory. Phys. Rev. Lett. 55, 2471–2474 (1985). 10032153 10.1103/PhysRevLett.55.2471

[b39] Lippert G., Hutter J. & Parrinello M. A hybrid Gaussian and plane wave density functional scheme. Mol. Phys. 92, 477–488 (1997).

[b40] VandeVondele J. & Hutter J. Gaussian basis sets for accurate calculations on molecular systems in gas and condensed phases. J. Chem. Phys. 127, 114105 (2007). 17887826 10.1063/1.2770708

[b41] Grimme S. Semiempirical GGA-type density functional constructed with a long-range dispersion correction. J. Comput. Chem. 27, 1787–1799 (2006). 16955487 10.1002/jcc.20495

[b42] Schmidt J. *et al.* Isobaric-isothermal molecular dynamics simulations utilizing density functional theory: an assessment of the structure and density of water at near-ambient conditions. J. Phys. Chem. B 113, 11959–11964 (2009). 19663399 10.1021/jp901990u

[b43] Kühne T. D., Pascal T. A., Kaxiras E. & Jung Y. New Insights into the structure of the vapor/water interface from large-scale first-principles simulations. J. Phys. Chem. Lett. 2, 105–113 (2011). 26295528 10.1021/jz101391r

[b44] Putrino A., Sebastiani D. & Parrinello M. Generalized variational density functional perturbation theory. J. Chem. Phys. 113, 7102 (2000).

[b45] Weber V. *et al.* Magnetic linear response properties calculations with the Gaussian and augmented-plane-wave method. J. Chem. Phys. 131, 014106 (2009). 19586095 10.1063/1.3156803

[b46] Lippert G., Hutter J. & Parrinello M. The Gaussian and augmented-plane-wave density functional method for ab initio molecular dynamics simulations. Theor. Chem. Acc. 103, 124–140 (1999).

[b47] Krack M. & Parrinello M. All-electron ab-initio molecular dynamics. Phys. Chem. Chem. Phys. 2, 2105–2112 (2000).

[b48] Kutzelnigg W., Fleischer U. & Schindler M. NMR Basic Principles and Progress Vol. 23, 165–262Springer (1991).

[b49] VandeVondele J. *et al.* Quickstep: fast and accurate density functional calculations using a mixed Gaussian and plane waves approach. Comput. Phys. Commun. 167, 103–128 (2005).

[b50] Kumar R., Schmidt J. R. & Skinner J. L. Hydrogen bonding definitions and dynamics in liquid water. J. Chem. Phys. 126, 204107 (2007). 17552754 10.1063/1.2742385

