# Supplementary material for: Covalency of hydrogen bonds in liquid water can be probed by proton nuclear magnetic resonance experiments
Source: Nat Commun. 2015 Sep 15;6:8318. doi: 10.1038/ncomms9318 (PMC4647854; doi:10.1038/ncomms9318)
Supplement: Supplementary Information — Supplementary Figures 1-3, Supplementary Tables 1-2 and Supplementary Note 1 [file ncomms9318-s1.pdf]

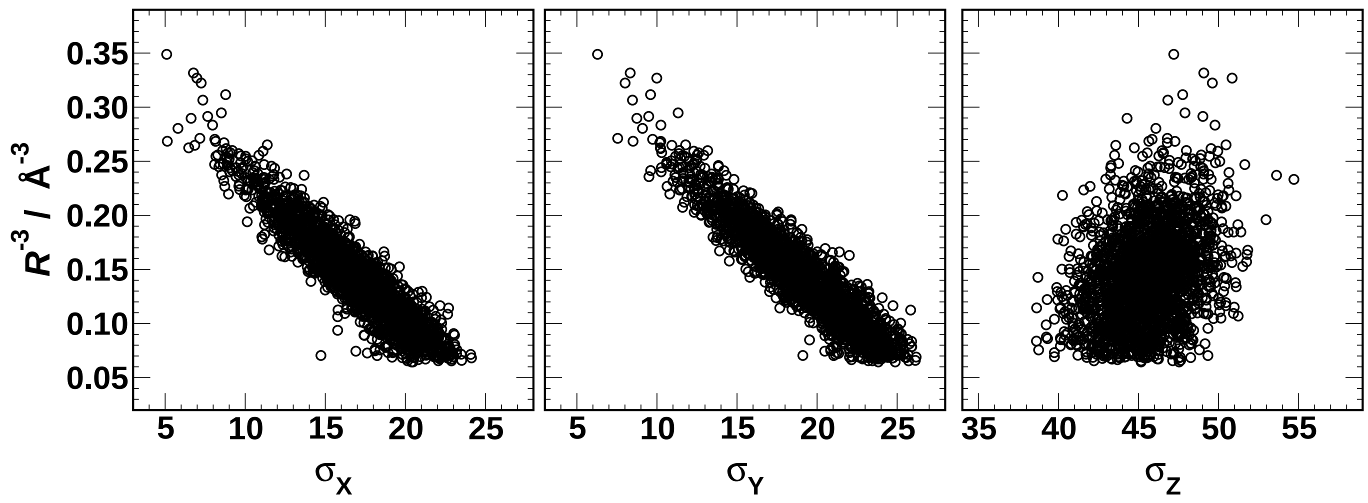

Supplementary Figure 1. Dependence of the three principal components of the shielding tensor on $\mathbf{R^{-3}}$. R is the hydrogen bond length as depicted in Figure 2.

As the supplementary Fig. 1 shows, we found that the components of the NMR shielding tensor σ depend differently on the intermolecular distance R between the hydrogen and oxygen atoms forming a HB. To explain the observed dependence we represent the Cartesian α-component of the induced magnetic field at the hydrogen atom as a multipolar series

$$\mathrm{B}_\alpha^{\mathrm{ind}}(R) = \mathrm{B}_\alpha^{\mathrm{ind}}(R \to \infty) + \frac{\mu_0}{4\pi} \sum_N \left[\left(\nabla_\alpha \nabla_\beta R_N^{-1}\right) \mathbf{M}_\beta^{(N)} - \frac{1}{3} \left(\nabla_\alpha \nabla_\beta \nabla_\gamma R_N^{-1}\right) \mathbf{M}_{\beta\gamma}^{(N)} + \ldots \right], \qquad (1)$$

where the N-summation is over all neighboring molecules in the system, while $\mathrm{M}_{\beta\gamma\ldots}$ are the components of the magnetic dipole, quadrupole and higher multipoles (supplementary Fig. 2). The term $\mathrm{B}_\alpha^{\mathrm{ind}}(R \to \infty)$ denotes the induced field originating from the water molecule carrying the hydrogen (at its instantaneously distorted and polarized state in bulk water).

$\boldsymbol{\sigma_\mathrm{X}}$ **and** $\boldsymbol{\sigma_\mathrm{Y}}$ **components.** In the case of $\sigma_\perp$, we observe a very strong linear dependence on R^{-3} of the closest electron-donor molecule: the Pearson correlation coefficients are $r = -0.928$ and -0.930 for σ_X and σ_Y, respectively. This is to to say that it suffices to keep only the closest water molecule in the N-summation and just the leading (dipolar) term in the multipole expansion. The corresponding induced field already accounts for more than 86% of the variance of σ_X and σ_Y. The negative correlation suggest a deshielding dipolar field from the acceptor water. Furthermore, the strong correlation implies that $\sigma_\perp$ is not influenced by the *intramolecular* geometry.

$\boldsymbol{\sigma_\mathrm{Z}}$ **component.** In the case of σ_Z the correlation with R is weak (though statistically significant $r = 0.327$) and the dipolar field is shielding. Nevertheless, it is still possible to keep only the contribution from the closest electron donor and discard the contributions from the other more distant water molecules. However, we found that a satisfactory regression model capable of predicting the behaviour of σ_Z should include at least three predictor variables: R^{-3}, the HB angle θ and the *intramolecular* O–H bond length $OH\text{-}r$ (supplementary Table 1). Thus, it appears that the σ_Z component is strongly influenced by the fields originating from the proton's own water molecule.

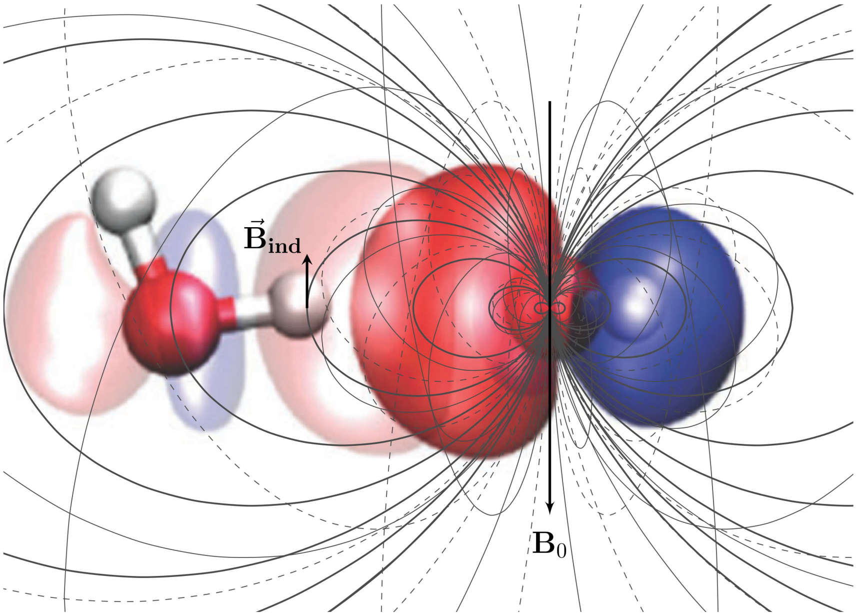

Supplementary Figure 2. The induced dipolar magnetic field at an electron donor water molecule. From supplementary Fig. 1, the induced magnetic field from the electron-donor water molecule can be very well-approximated by a dipolar magnetic field originating from the position of the oxygen atom. The field is shielding along the HB axis and deshielding in the orthogonal plane.

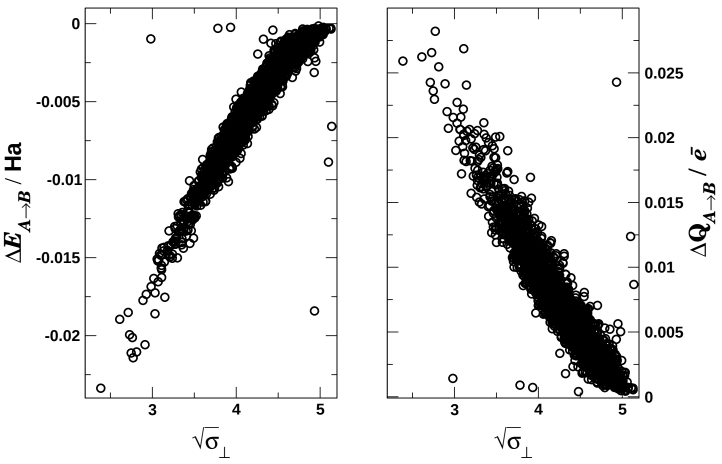

Supplementary Figure 3. Quadratic dependence between electronic parameters and $\sigma_\perp$.

	R^{-3}	$\cos\theta$	OH-r	$\mathcal{R}^2$
σ_Z	1.088	-0.645	-1.937	0.838
$\sigma_\perp$	-2.606	0.329	-0.595	0.931

Supplementary Table 1. Standardized multiple regression coefficients for σ_Z and $\sigma_\perp$. R is the HB length, θ the HB angle, OH-r the covalent O-H bond length and $\mathcal{R}^2$ the adjusted coefficient of determination.

The regression coefficients are obtained as a set of parameters $a_{\{\alpha\}}$ and b that minimize the sum of squared residuals $\sum_{i=1}^{P}(y_i - \sigma_i)^2$, where σ_i are the data points being predicted and $\eta_i = \sum_\alpha a_\alpha x_{\alpha,i} + b$ is a linear combination of the predictor variables x_i. The standardized regression coefficients are obtained after standardizing all predictor variables x_α so that each has zero mean and unit standard deviation.

	a	b	R^2
$\Delta Q_{A\to B}$	-0.01 ($\pm$7E-05)	0.049($\pm$3E-04)	0.88
$\Delta E_{A\to B}$	0.008 ($\pm$4E-05)	-0.038($\pm$2E-04)	0.91

Supplementary Table 2. Least-squares regression coefficients for the phenomenological model relating the electronic parameters to $\sigma_\perp$. See supplementary Note 1.

Supplementary Note 1: Calculation of averages of electron transfer descriptors from the averages of the NMR tensor components

In our paper, we calculate the ensemble averaged intermolecular electron transfer $\langle \Delta Q_{A\to B}\rangle$ (and its energy $\langle \Delta E_{A\to B}\rangle$) from the experimentally measured ensemble average of $\langle \sigma_\perp \rangle$. As Figure 1 and Equation 3 show, the dependence between these quantities is not linear and, therefore, care must be taken to compute the average of one quantity from the average of the other. This section describes how this is done.

Here, we denote $\Delta Q_{A\to B}$ as Q for simplicity. Q is a function of $\sigma_\perp$ and we expand it in a Taylor series around $\langle \sigma_\perp \rangle$:

$$Q(\sigma_\perp) \;=\; Q(\langle \sigma_\perp \rangle) + Q'(\langle \sigma_\perp \rangle) \cdot (\sigma_\perp - \langle \sigma_\perp \rangle) + Q''(\langle \sigma_\perp \rangle) \cdot (\sigma_\perp - \langle \sigma_\perp \rangle)^2 + \ldots \tag{2}$$

Using the fact that $\langle \sigma_\perp - \langle \sigma_\perp \rangle \rangle = 0$, the average of $Q(\sigma_\perp)$ is given by

$$\langle Q(\sigma_\perp) \rangle \;=\; Q(\langle \sigma_\perp \rangle) + Q''(\langle \sigma_\perp \rangle) \cdot \text{var}(\sigma_\perp) + \ldots \tag{3}$$

The dependence of Q on $\sigma_\perp$ is almost linear (Figure 1). Therefore, it is reasonable to expect that it is sufficient to estimate the second derivative term $Q''(\langle \sigma_\perp \rangle)$ and neglect higher order terms. The second derivative can be estimated using either the raw data in Figure 1, or the derived function given by Equation 3. Here, the first approach is selected.

In order to obtain a numerical estimate of the second derivative, we represented the quadratic dependence between variables as $Q = a\sqrt{\sigma_\perp} + b$ (supplementary Fig. 3 confirms this choice) and obtained numerical values of parameters a and b using the standard least squares fitting procedure. The fitted coefficients are shown in supplementary Table 2 for both charge $\Delta Q_{A\to B}$ and energy $\Delta E_{A\to B}$.

The Taylor series can now be written as

$$\langle Q(\sigma_\perp) \rangle \;=\; Q(\langle \sigma_\perp \rangle) - \frac{a}{4} \langle \sigma_\perp \rangle^{-3/2} \cdot \text{var}(\sigma_\perp) + \cdots \tag{4}$$

and gives the second-order correction of 0.37 milli-electron for Q and -0.29 mHa for E. The corrections are small compared to the corresponding first order terms in the Taylor expansion and thus can be neglected.
